# In this issue

**Published:** 2022-11

**The impact of COVID-19 on children and adolescents’ mental health**

Hafiz & Aljadani address the mental and social health needs of children and adolescents after the pandemic. Children and adolescents are more susceptible to the formation of mental health issues, since their brains are still developing. There are still issues to be resolved to give children and adolescents in many regions of the world with quality, rights-based, and culturally relevant mental health care. It is essential to understand the extent of the issue to devise effective strategies and interventions with which to tackle it. Such measures can deal with fears, helping to develop positive psychological behaviors which can lead children and adolescents to overcome such an unprecedented situation and its associated stressors.

***see page 1183***

