# Supplementary material for: In this issue
Source: Saudi Med J. 2022 Nov;43(11):1181. (PMC10043920)
Supplement: Related texts

# Abstract: Original Articles

**The role of intra-articular injection of autologous platelet-rich plasma versus corticosteroids in the treatment of synovitis in lumbar facet joint disease**

Kotb et al compare the efficacy between platelet-rich plasma (PRP) and corticosteroids (CS) in improving magnetic resonance imaging (MRI)-detected synovitis in correlation with clinical complaints among patients with lumbar facet joint (FJ) disease. A prospective, randomized, comparative, single blinded study included 30 patients with lumbar FJ disease, divided into 2 equal groups, received PRP and CS injections. Both groups showed a significant improvement in all mentioned parameters at follow-up after 3 months. Both PRP and CS injections were effective in improving MRI-detected FJ synovitis while concurrently improving all examined parameters at follow-up after 3 months.

***see page 1200***

**National research guideline for prehospital emergency medical care. *A prospective Delphi-study***

**Figure:** Flow chart of the study process.

Alghamdi et al identify 100 prehospital experts in Saudi Arabia were invited to participate in all 3 rounds. Of these, 47 responded in round I, 34 in round II, and 39 in round III. In round I, participants submitted 278 research topics. After deduplication and sorting, 78 topics were assessed in the other 2 rounds. The study was carried out between November 2021 and February 2022. A 3-round modified Delphi consensus methods were used to determine high-priority research topics. Round I included an open-ended question to list all high-priority research topics in a prehospital setting in Riyadh, Saudi Arabia. Rounds II and III included ranking evaluation and consensus agreement. e included topics were listed based on the agreement of ≥70% of the experts participating in the study. e study’s implication is to determine research priorities which will be provided to the Saudi Research, Development and Innovation Authority, Ministry of Health, Saudi Red Crescent Authority, and research centers to disseminate our results, apprise future prehospital research plans, and prioritize funding. Overall, the results of this study can significantly improve the care and quality of emergency medical service.

***see page 1265***

# Abstract: CASE REPORT

**Acute myocarditis after COVID-19 vaccination**

**Figure:** Echocardiography on the first day of admission; left ventricle size and wall thickness.

Saadi et al evaluate the complications that faced an 18-year-old male who received a COVID-19 vaccine and diagnosed with myocarditis/ pericarditis. He was complaining of a severe diffuse chest pain for 5 hours that prevented him from sleeping and there is change in breath and standing, without dyspnea or palpitation. He denied any recent viral illness. He never had history of any drug intake or previous surgeries. He felt these drawbacks only after receiving COVID-19 vaccination on the 5th of February 2022.

***see page 1270***
